# Reduction in short interval intracortical inhibition from the early stage reflects the pathophysiology in amyotrophic lateral sclerosis: A meta‐analysis study

**DOI:** 10.1111/ene.16281

**Published:** 2024-03-20

**Authors:** Mana Higashihara, Nathan Pavey, Parvathi Menon, Mehdi van den Bos, Kazumoto Shibuya, Satoshi Kuwabara, Matthew C. Kiernan, Masayoshi Koinuma, Steve Vucic

**Affiliations:** ^1^ Department of Neurology Tokyo Metropolitan Institute for Geriatrics and Gerontology Tokyo Japan; ^2^ Brain and Nerve Research Center University of Sydney Sydney New South Wales Australia; ^3^ Neurology, Graduate School of Medicine Chiba University Chiba Japan; ^4^ Neuroscience Resarch Australia University of New South Wales Sydney New South Wales Australia; ^5^ Department of Neurology Royal Prince Alfred Hospital Sydney New South Wales Australia; ^6^ Faculty of Pharmaceutical Sciences Teikyo Heisei University Tokyo Japan; ^7^ Healthy Aging Innovation Center Tokyo Metropolitan Institute for Geriatrics and Gerontology Tokyo Japan

**Keywords:** amyotrophic lateral sclerosis, cortical hyperexcitability, motor neuron disease, SICI, threshold tracking TMS

## Abstract

**Background and purpose:**

Cortical hyperexcitability has been identified as a diagnostic and pathogenic biomarker of amyotrophic lateral sclerosis (ALS). Cortical excitability is assessed by transcranial magnetic stimulation (TMS), a non‐invasive neurophysiological technique. The TMS biomarkers exhibiting highest sensitivity for cortical hyperexcitability in ALS remain to be elucidated. A meta‐analysis was performed to determine the TMS biomarkers exhibiting the highest sensitivity for cortical hyperexcitability in ALS.

**Methods:**

A systematic literature review was conducted of all relevant studies published in the English language by searching PubMed, MEDLINE, Embase and Scopus electronic databases from 1 January 2006 to 28 February 2023. Inclusion criteria included studies reporting the utility of threshold tracking TMS (serial ascending method) in ALS and controls.

**Results:**

In total, more than 2500 participants, incorporating 1530 ALS patients and 1102 controls (healthy, 907; neuromuscular, 195) were assessed with threshold tracking TMS across 25 studies. Significant reduction of mean short interval intracortical inhibition (interstimulus interval 1–7 ms) exhibited the highest standardized mean difference with moderate heterogeneity (−0.994, 95% confidence interval −1.12 to −0.873, *p* < 0.001; *Q* = 38.61, *p* < 0.05; *I* ^2^ = 40%). The reduction of cortical silent period duration along with an increase in motor evoked potential amplitude and intracortical facilitation also exhibited significant, albeit smaller, standardized mean differences.

**Conclusion:**

This large meta‐analysis study disclosed that mean short interval intracortical inhibition reduction exhibited the highest sensitivity for cortical hyperexcitability in ALS. Combined findings from this meta‐analysis suggest that research strategies aimed at understanding the cause of inhibitory interneuronal circuit dysfunction could enhance understanding of ALS pathogenesis.

## INTRODUCTION

Amyotrophic lateral sclerosis (ALS) is a rapidly progressive and fatal neurodegenerative disorder characterized by progressive upper and lower motor neuron dysfunction [1]. Cortical hyperexcitability has been identified as an early and intrinsic feature of ALS [2, 3, 4], mediated through a combination of cortical disinhibition and an increase in facilitation [5, 6, 7]. At a clinical level, cortical hyperexcitability may manifest as hyper‐reflexia and spasticity [8], potentially accounting for the split‐hand phenomenon as well as concordance between handedness and disease‐onset site [9, 10]. Cortical hyperexcitability has been consistently identified as a pathogenic and diagnostic biomarker of ALS [7], preceding lower motor neuron dysfunction [3, 7] and contributing to disease evolution and adverse prognosis [9, 11].

Transcranial magnetic stimulation (TMS) is a non‐invasive technique for assessing cortical motor function [12]. Paired‐pulse TMS assesses cortical interneuronal function by delivering a modulating subthreshold conditioning stimulus prior to a test stimulus. The original ‘constant‐stimulus’ method measured the reduction in the test stimulus motor evoked potential (MEP) amplitude at an interstimulus interval (ISI) of 1–5 ms, termed short interval intracortical inhibition (SICI), a biomarker of cortical inhibitory interneuronal circuits acting via γ‐aminobutyric acid type A (GABA_A_) receptors [12, 13]. Subsequently, a threshold tracking technique was developed to overcome the potential limitation of MEP variability, whereby SICI was heralded by greater conditioned‐test stimulus intensity required to generate and maintain the target MEP response of fixed amplitude (0.2 mV ± 20%) [14, 15]. The reduction of SICI has been consistently identified as a biomarker of cortical hyperexcitability in ALS with diagnostic and pathogenic implications [2, 3, 4, 9, 10, 11, 16, 17, 18]. An increase in intracortical facilitation (ICF), between ISIs 10–30 ms, has also been reported as a biomarker of cortical hyperexcitability in ALS [7]. The combination of reduction in SICI along with an increase in ICF and MEP amplitude as well as reduction in resting motor threshold results in an imbalance between cortical inhibition and facilitation leading to hyperexcitability in ALS [18].

Single‐pulse TMS has yielded additional biomarkers of cortical hyperexcitability in ALS [5, 9]. Specifically, reduction of cortical silent period duration has been reported as a biomarker of cortical hyperexcitability in ALS and reflects dysfunction of long latency GABAergic inhibitory circuits acting via GABA_B_ receptors [2, 16, 17, 19, 20]. Increased MEP amplitude has also been a well described biomarker of cortical dysfunction in ALS, indicating enhanced corticomotoneuronal glutamatergic activity [3, 7, 10, 16, 17, 20]. Reduction in resting motor threshold (RMT) has been described in ALS, although conflicting results have been reported, with some studies reporting normal [2, 3, 9, 21] or even increased RMTs [22, 23].

Transcranial magnetic stimulation (TMS) derived measures of cortical motor function could be of clinical utility in ALS, potentially serving as a surrogate diagnostic and prognostic biomarker, although it remains to be determined which of these biomarkers is of greatest clinical utility. Whilst reduction of mean SICI (between ISIs 1–7 ms) was reported to be the most sensitive TMS measure in ALS [7], replication in larger patient cohorts is required. Consequently, the aim of this meta‐analysis was to investigate which TMS‐derived biomarker of cortical motor function exhibited the greatest utility in differentiating ALS from controls, and to determine the impact of ALS disease duration, functional status and disease‐onset site on clinical utility.

## MATERIALS AND METHODS

### Literature search

The first study reporting the clinical utility of threshold tracking TMS in ALS was published in 2006 [3]. Consequently, a systematic literature review was conducted of all relevant studies published in the English language by searching PubMed, MEDLINE, Embase and Scopus electronic databases from 1 January 2006 to 28 February 2023. The search for eligible studies to be included in the meta‐analysis was conducted in accordance with the Preferred Reporting Items for Systematic Reviews and Meta‐Analyses (PRISMA) guidelines [24]. The search strategy comprised keywords and MeSH terms, in isolation or combination, and included ‘amyotrophic lateral sclerosis’, ‘cortical hyperexcitability’, ‘motor neuron disorders’, ‘neurodegenerative disease’, ‘neuromuscular disease’, ‘primary lateral sclerosis’, ‘progressive muscular atrophy’, ‘threshold tracking transcranial magnetic stimulation’ or ‘TMS’. The meta‐analysis was registered in PROSPERO (ID#: CRD42023428881).

### Data collection and quality assessment

The inclusion criteria included (i) studies reporting threshold tracking TMS using the serial ascending method [14] in ALS and controls, also referred to as the Sydney method by Tankisi et al. [25]; and (ii) studies reporting at least one threshold tracking TMS parameter, SICI. In addition, the use of a 90‐mm circular coil and recording from the abductor pollicis brevis muscle was an additional criterion.

Exclusion criteria included (i) studies reporting constant stimulus or parallel threshold tracking TMS methods; (ii) case reports, reviews, letters or editorial; (iii) conference abstracts; (iv) small studies (sample size <10); or (v) studies reporting the same ALS patients and/or control cohorts. A concerted effort was undertaken to avoid duplication of subjects (ALS patients and controls) across the included papers, and due to this concern a number of potential papers were excluded from the current meta‐analysis [26, 27, 28, 29, 30, 31, 32, 33]. Additionally, the number of subjects included for each study is depicted in Table 1. Two authors (MH and SV) independently reviewed eligible studies to ensure fulfilment of inclusion and exclusion criteria, and a final list of studies was assembled based on author consensus. The Newcastle–Ottawa Scale was used to examine study quality. Data collection was based on published studies only and consequently informed consent and ethical committee review were not required.

**TABLE 1:** Summary of studies included in the meta‐analysis with the number of amyotrophic lateral sclerosis (ALS) patients and controls.

Study	Year	ALS	Controls
Suzuki et al. [34]	2022	53	30
Higashihara et al. [35]	2021	40	42
Agarwal et al. [36]	2021	39	30
van den Bos et al. [37]	2021	17	18
Menon et al.^a^ [38]	2020	345	99
Dharmadasa et al. [11]	2020	138	114
Menon et al.^a^ [39]	2019	60	28
van den Bos et al. [40]	2018	27	25
Menon et al. [9]	2017	50	45
Geevasinga et al. [20]	2017	20	11
Matamala et al. [41]	2017	21	40
Shibuya et al. [42]	2016	169	109
Geevasinga et al. [43]	2016	19	31
Menon et al. [44]	2016	18	60
Grieve et al. [45]	2016	25	30
Geevasinga et al. [17]	2015	15	74
Menon et al. [46]	2015	24	33
Menon et al.^a^ [7]	2015	209	68
Menon et al. [10]	2014	26	21
Geevasinga et al. [47]	2014	82	34
Bae et al. [48]	2014	17	15
Vucic et al. [49]	2013	25	30
Vucic et al. [4]	2008	57	55
Vucic et al. [16]	2007	11	26
Vucic et al. [3]	2006	23	34

^a^ All controls were ALS mimics, whilst the remainder were healthy controls.

The TMS outcome biomarkers included the difference in mean SICI between ISIs 1–7 ms between ALS patients and controls, as well as differences in mean ICF (ISI 10–30 ms), MEP amplitude (expressed as percentage of compound muscle action potential), maximum cortical silent period (CSP) duration (ms) and RMT (% maximum stimulator output).

Clinical features were also collated and included the ALS Functional Rating Scale Revised (ALSFRS‐R) [50], disease duration (months), site of disease onset and upper motor neuron (UMN) score [51]. The UMN score includes biceps brachii, triceps, supinator, finger, patellar, ankle reflexes and plantar flexor reflexes measured on both sides, along with facial and jaw jerks with the score ranging from 0 (no UMN signs) to a maximum score of 16, signifying severe UMN signs [51].

### Statistical analysis

#### Meta‐analysis

Standardized mean differences (SMDs, Cohen's *d*), standard error and 95% confidence intervals (CIs) were estimated for TMS parameters recorded for ALS patients and controls. Random‐effects models were used to determine SMD. Heterogeneity was assessed using the Higgins index (*I* ^2^). Publication bias was assessed by using a funnel plot, Egger's weighted regression method [52] and Duval and Tweedie's trim and fill method [53].

#### Meta‐regression

Meta‐regression analyses were performed to confirm the association between TMS measures and the following clinical parameters: (i) duration of disease (months), (ii) ALSFRS‐R, (iii) site of disease onset, (iv) gender and (v) UMN score. Meta‐regression analysis was performed with a random‐effects model using restricted maximum likelihood estimation. Given the exploratory purposes of these analyses, combinations were selected for which the *p* value of the regression coefficient of the clinical parameters was less than or equal to 0.1 and in keeping with previous meta‐analyses [54, 55]. All other data are expressed as mean ± standard deviation or median (interquartile range), with *p* values <0.05 considered statistically significant.

## RESULTS

### Study selection and characteristics

**FIGURE 1:**
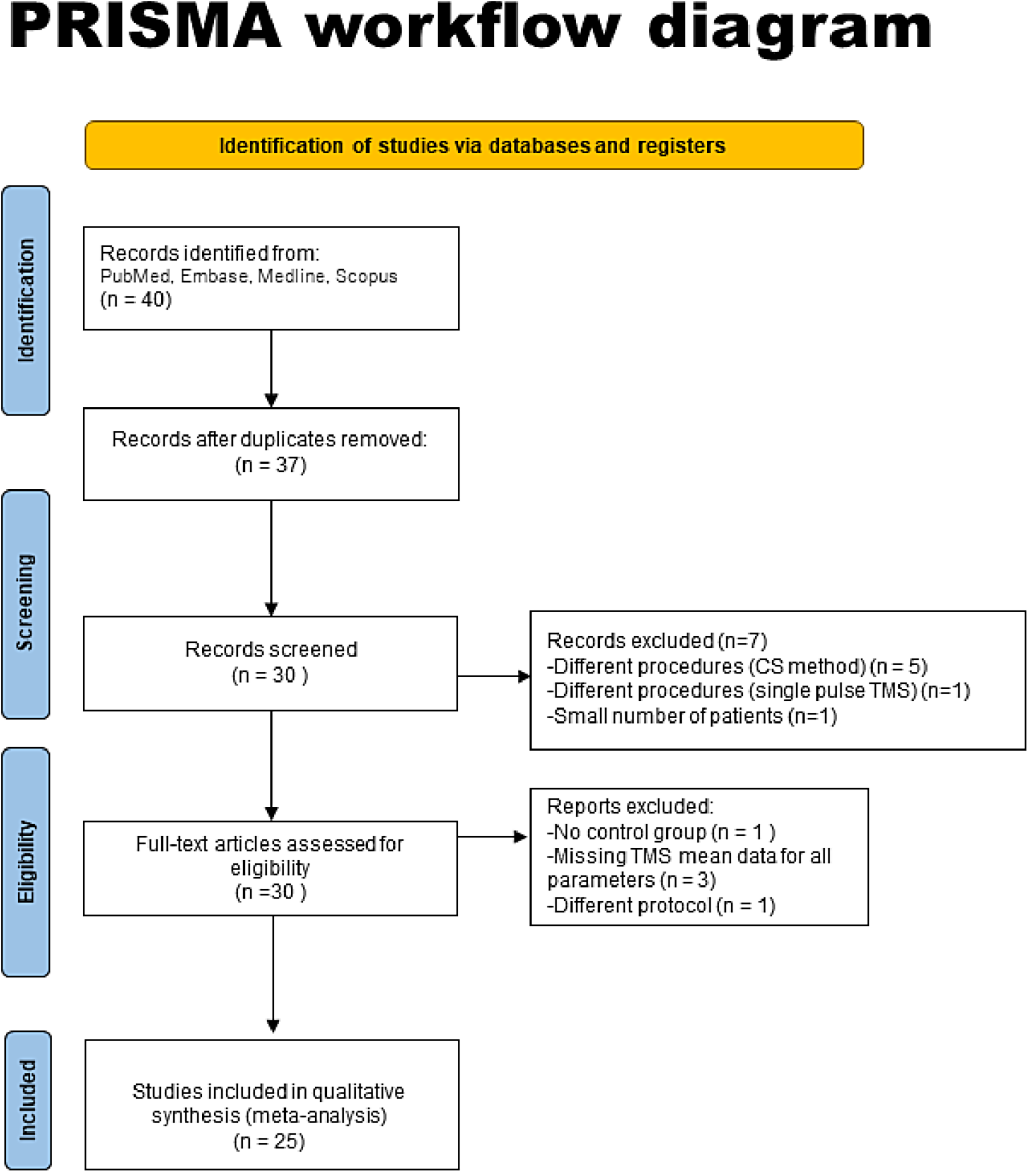
PRISMA flow diagram detailing the bibliographical searches carried out, including the number of papers screened and the reasons for exclusion of papers from further analysis.

The study selection process is described in the flow diagram (Figure 1). During the initial search, 40 studies were identified from the PubMed, MEDLINE, Embase and Scopus databases. After a screening and eligibility criteria assessment 25 studies met the inclusion criteria. The Newcastle–Ottawa Scale score was ≥7 for all included studies indicating high quality.

In total, 1530 ALS patients and 1102 controls (healthy, 907; neuromuscular, 195) were assessed with threshold tracking TMS in the 25 studies. Demographic, clinical and TMS characteristics of all participants are summarized in Table 2. In ALS, limb‐onset disease was evident in 69% and bulbar‐onset disease in 31% of patients. The median ALSFRS‐R score was 42 (40.7–42.8) indicating moderate functional disability, whilst the median UMN score was 12 (10.8–12) being consistent with the presence of prominent UMN signs. Additionally, median disease duration in ALS patients was 15.3 (11–17.5) months, indicating that assessment occurred in the early stages of the disease. Sixteen studies reported on riluzole use (*N* = 1150), with 691 (60%) ALS patients reportedly receiving treatment at time of TMS testing. Apart from riluzole therapy, the use of medications that could impact on TMS findings was not reported in the included studies.

**TABLE 2:** Summary of clinical and transcranial magnetic stimulation (TMS) characteristics of amyotrophic lateral sclerosis (ALS) patients and controls.

	ALS	Controls
Number of participants	1530	1102
Median age at assessment (years)	60.7	53
(IQR)	(59–63)	(48–58)
Males:females	933:601	530:437
Median disease duration (months)	15.3	
(IQR)	(11–17.5)	
Site of disease onset		
Limb	68.6%	
Bulbar	31.4%	
Median ALSFRS‐R	42	
(IQR)	(40.7–42.8)	
Median UMN score	12	
(IQR)	(10.8–12)	
Mean SICI between ISIs 1 and 7 ms (%)	2.7	11.1
(SD)	(8.7)	(6.7)
Mean CSP duration (ms)	179.2	204.8
(SD)	(44.6)	(32.7)
Mean ICF (%)	−2.3	−0.5
(SD)	(6.8)	(5.3)
Mean MEP amplitude (%)	37.5	26.3
(SD)	(26.3)	(17)
Mean RMT (%)	58.3	58.8
(SD)	(11.9)	(9.8)

*Note*: The average of the mean TMS parameters is reported. The control population comprised healthy controls and patients with neuromuscular mimicking disorders including Kennedys' disease, autoimmune neuropathies (chronic inflammatory demyelinating polyradiculoneuropathy, multifocal motor neuropathy), acquired neuromyotonia, facial onset sensory and motor neuronopathy syndrome, spinal muscular atrophy, Hirayama disease, myasthenia gravis. All data are expressed as mean (standard deviation, SD) or median (interquartile range, IQR).

Abbreviations: ALSFRS‐R, Amyotrophic Lateral Sclerosis Functional Rating Scale Revised; CSP, cortical silent period; ICF, intracortical facilitation; ISI, interstimulus interval 1–7 ms; MEP amplitude, motor evoked potential amplitude expressed as a percentage of the compound muscle action potential amplitude; RMT, resting motor threshold; SICI, short interval intracortical inhibition; UMN, upper motor neuron.

### Measures of cortical inhibition

#### Short interval intracortical inhibition

**FIGURE 2:**
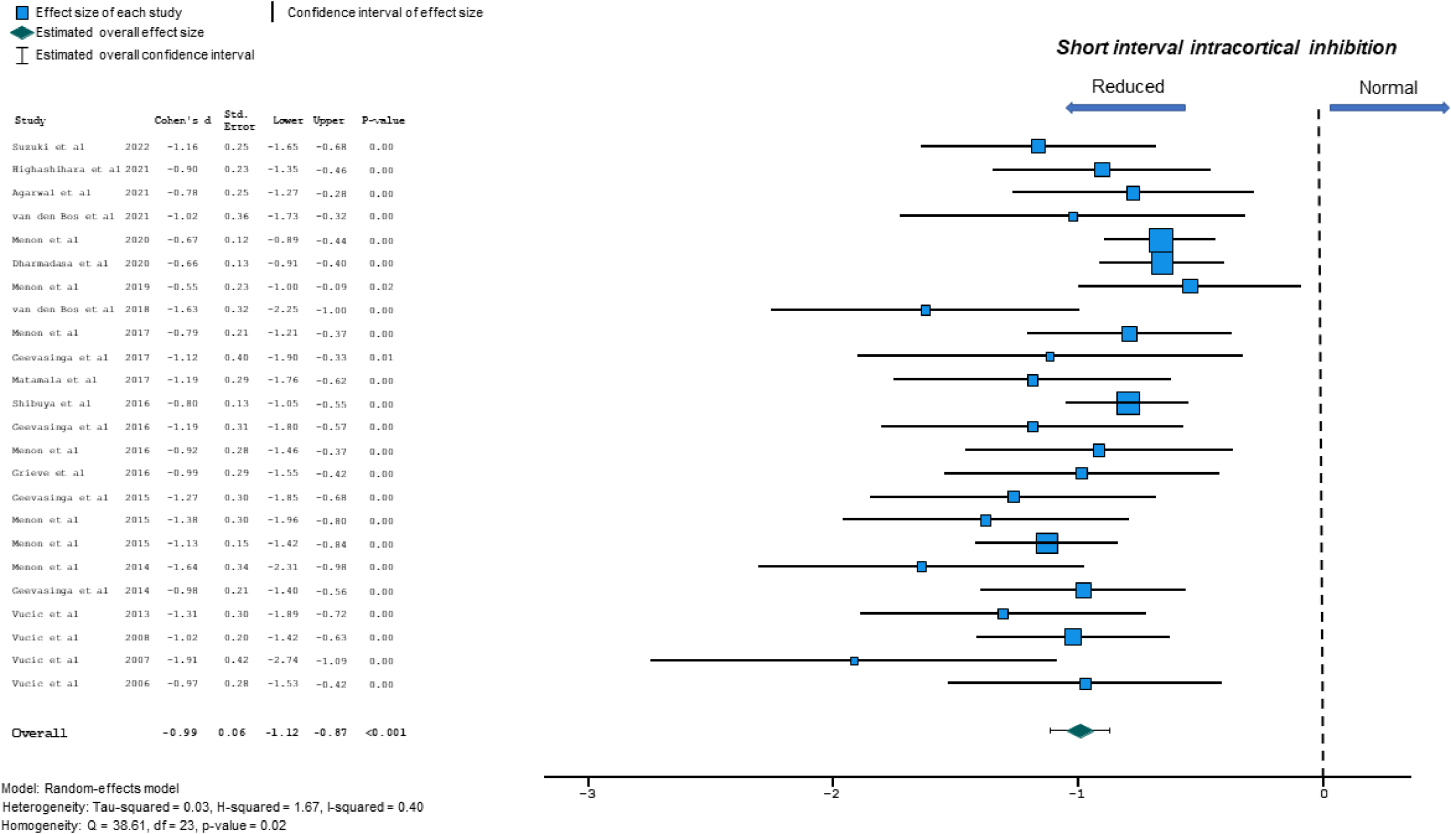
Forest plot depicting effect sizes for individual studies and an overall effect size for short interval intracortical inhibition (SICI). The dotted line represents an absence of significant difference in SICI between amyotrophic lateral sclerosis patients and controls.

Reduction in mean SICI, between ISI 1–7 ms, has been reported as a robust biomarker of cortical hyperexcitability in ALS [7]. Consequently, all selected studies reported differences in mean SICI (ISI 1–7 ms) between ALS patients and controls. A significant reduction of mean SICI was reported in ALS patients with evidence of moderate heterogeneity (SMD −0.99, 95% CI −1.12 to −0.87, *p* < 0.001; *Q* = 38.61, *p* < 0.05; *I* ^2^ = 40%; Figure 2). Significant funnel plot asymmetry was evident when assessed by Egger's test with an estimated intercept value being −0.44 (*p* = 0.002). Importantly, the trim and fill method indicated that ‘study imputation’ did not exert significant effects on SMD (−0.854, 95% CI −0.987 to −0.772, *p* < 0.0001), suggesting that publication bias was not a concern.

Meta‐regression analysis suggested that disease duration, ALSFRS‐R, site of disease onset, UMN score or gender (proportion of males) were not significant predictors of the relationship between mean SICI and ALS (Table 3).

**TABLE 3:** Random effects meta‐regression.

	Disease duration (months)	ALSFRS‐R	Site of disease onset	UMN score	Gender
SICI					
Coefficient	−0.008	0.030	0.004	−0.009	−0.794
Standard error	0.008	0.050	0.004	0.031	1.415
95% CI_LOWER_	−0.020	−0.06	−0.010	−0.070	−3.567
95% CI_UPPER_	0.008	0.130	0.012	0.051	1.980
*Z* value	−1.01	0.71	0.85	−0.310	−0.56
*p* value	0.314	0.478	0.396	0.759	0.575
CSP duration					
Coefficient	−0.003	0.021	−0.004	−0.004	−1.630
Standard error	0.009	0.057	0.005	0.042	1.715
95% CI_LOWER_	−0.021	−0.090	−0.014	−0.085	−4.991
95% CI_UPPER_	0.015	0.132	0.007	0.078	1.731
*Z* value	0.340	0.370	−0.730	−0.090	−0.950
*p* value	0.734	0.714	0.467	0.931	0.342
ICF					
Coefficient	−0.014	0.038	0.006	−0.009	−2.404
Standard error	0.008	0.053	0.005	0.046	1.666
95% CI_LOWER_	−0.030	−0.066	−0.005	−0.098	−5.670
95% CI_UPPER_	0.003	0.142	0.016	0.081	0.862
*Z* value	−1.65	0.72	1.06	−0.190	−1.44
*p* value	0.099	0.471	0.289	0.852	0.149
MEP amplitude					
Coefficient	0.005	−0.063	−0.002	−0.020	−1.787
Standard error	0.008	0.051	0.005	0.042	1.612
95% CI_LOWER_	−0.011	−0.163	−0.012	−0.102	−4.948
95% CI_UPPER_	0.021	0.037	0.007	0.062	1.373
*Z* value	0.62	−1.23	−0.51	−0.480	−1.11
*p* value	0.54	0.22	0.61	0.631	0.27
RMT					
Coefficient	0.010	−0.088	−0.010	0. 037	1.141
Standard error	0.010	0.063	0.006	0.042	2.176
95% CI_LOWER_	−0.010	−0.211	−0.022	−0.046	−3.124
95% CI_UPPER_	0.030	0.035	0.002	0.120	5.406
*Z* value	1.000	−1.400	−1.690	0.870	0.52
*p* value	0.32	0.16	**0.09**	0.382	0.60

*Note*: The impact of disease duration from symptom onset, ALSFRS‐R, site of disease onset (limb vs. bulbar) and gender (male vs. female) on TMS parameters was assessed. Given the exploratory purposes of the meta‐regression analysis, combinations were selected for which the *p* value of the regression coefficient of the clinical parameters was ≤0.1.

Abbreviations: ALSFRS‐R, Amyotrophic Lateral Sclerosis Functional Rating Scale Revised; CI, confidence interval; CSP, cortical silent period; ICF, intracortical facilitation; MEP amplitude, motor evoked potential amplitude expressed as a percentage of the compound muscle action potential amplitude; RMT, resting motor threshold; SICI, short interval intracortical inhibition; TMS, transcranial magnetic stimulation; UMN, upper motor neuron.

#### Cortical silent period duration

**FIGURE 3:**
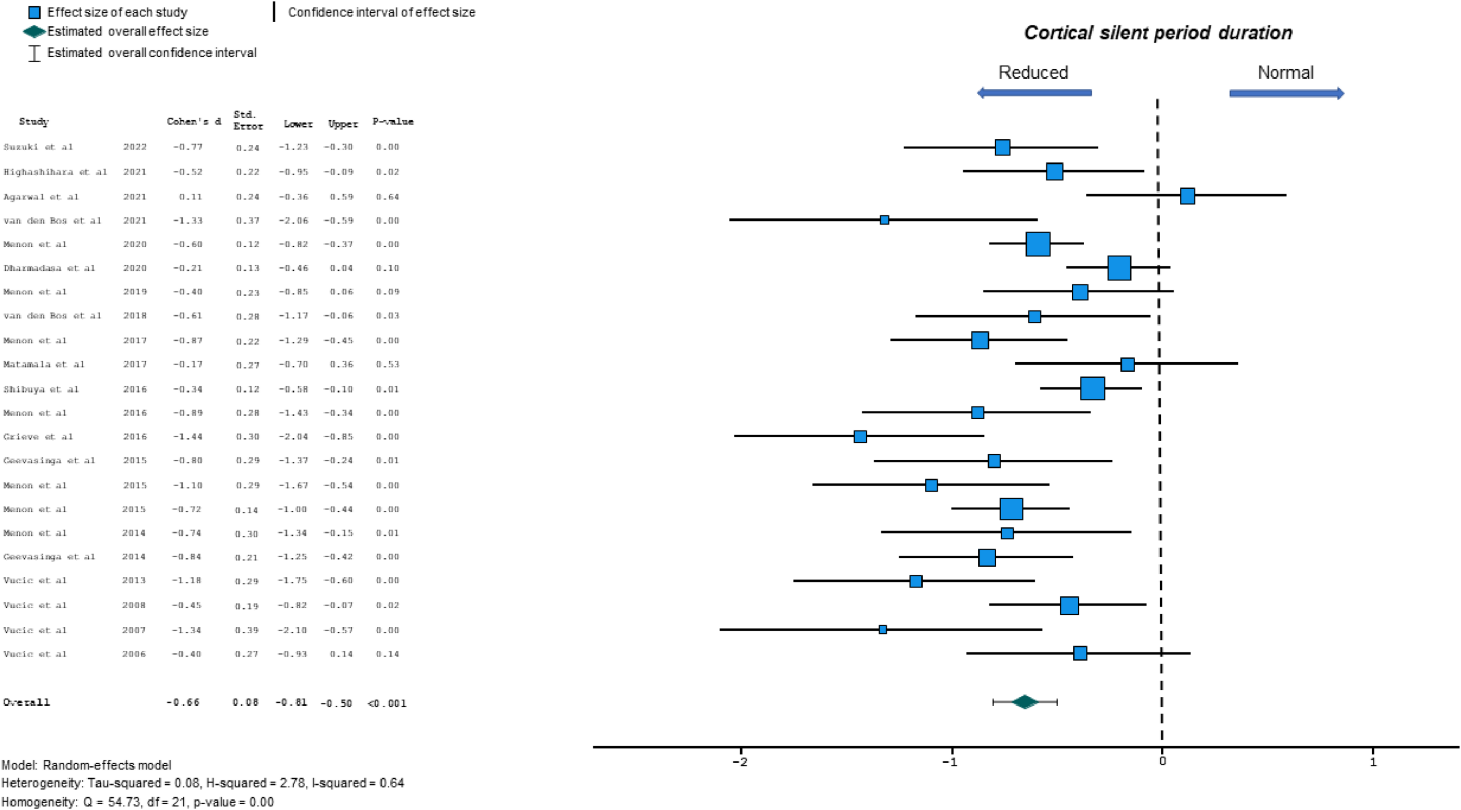
Forest plot depicting effect sizes for individual studies and an overall effect size for cortical silent period (CSP) duration. The dotted line represents an absence of significant difference in CSP duration between amyotrophic lateral sclerosis patients and controls.

A reduction in CSP duration has also been previously reported in ALS [7]. Meta‐analysis disclosed that reduced CSP duration was significantly associated with ALS with evidence of heterogeneity (−0.66, 95% CI −0.81 to −0.50, *p* < 0.001; *Q* = 54.73, *p* < 0.001; *I* ^2^ = 64%; Figure 3). Of relevance, the magnitude of effect size was less for CSP duration compared to the effect size for SICI. There was no significant funnel plot asymmetry (Egger's test estimated intercept −0.05, *p* = 0.81).

Meta‐regression analysis suggested that disease duration, ALSFRS‐R, site of disease onset, UMN score or gender (proportion of males) were not significant predictors of the relationship between mean CSP duration and ALS (Table 3).

### Measures of cortical facilitation

#### Intracortical facilitation

**FIGURE 4:**
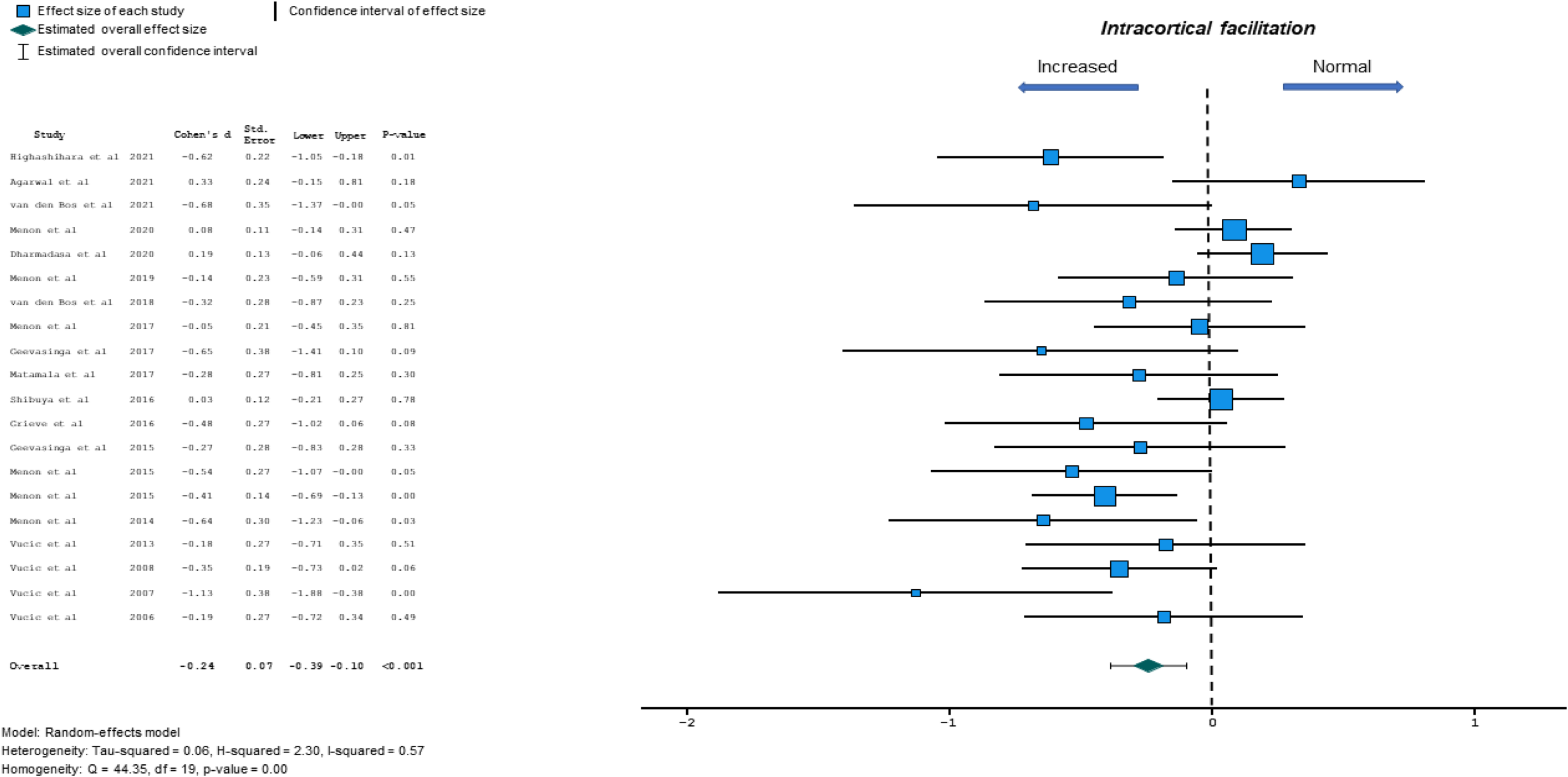
Forest plot depicting effect sizes for individual studies and an overall effect size for intracortical facilitation (ICF). The dotted line represents an absence of significant difference in ICF between amyotrophic lateral sclerosis patients and controls.

An increase in ICF (between ISIs 10 and 30 ms) has been previously reported in ALS patients, being represented by a greater mean negative ICF value [7]. Meta‐analysis revealed a significant increase of ICF in ALS patients compared to controls (−0.24, 95% CI −0.39 to −0.10, *p* < 0.001; *Q* = 44.35, *p* < 0.001; *I* ^2^ = 57%; Figure 4), with evidence of heterogeneity. There was no significant funnel plot asymmetry as assessed by Egger's test with an estimated intercept value being 0.35 (*p* = 0.06).

Meta‐regression analysis disclosed that disease duration was potentially related to mean ICF changes in ALS (*p* = 0.1, Table 3). Consequently, a subgroup analysis was undertaken which disclosed a trend for ICF increase in ALS patients with longer disease duration (SMD_>18 months from onset_ −0.37, 95% CI −0.95 to 0.22; SMD_≤18 months from onset_ −0.24, 95% CI −0.4 to −0.99). There was a high degree of heterogeneity (*I* ^2^ = 79.3%) in ALS patients exhibiting longer disease duration. In contrast, ALSFRS‐R, site of disease onset, UMN score or gender (proportion of males) were not significant predictors of a relationship between mean ICF and ALS (Table 3).

#### Motor evoked potential amplitude

**FIGURE 5:**
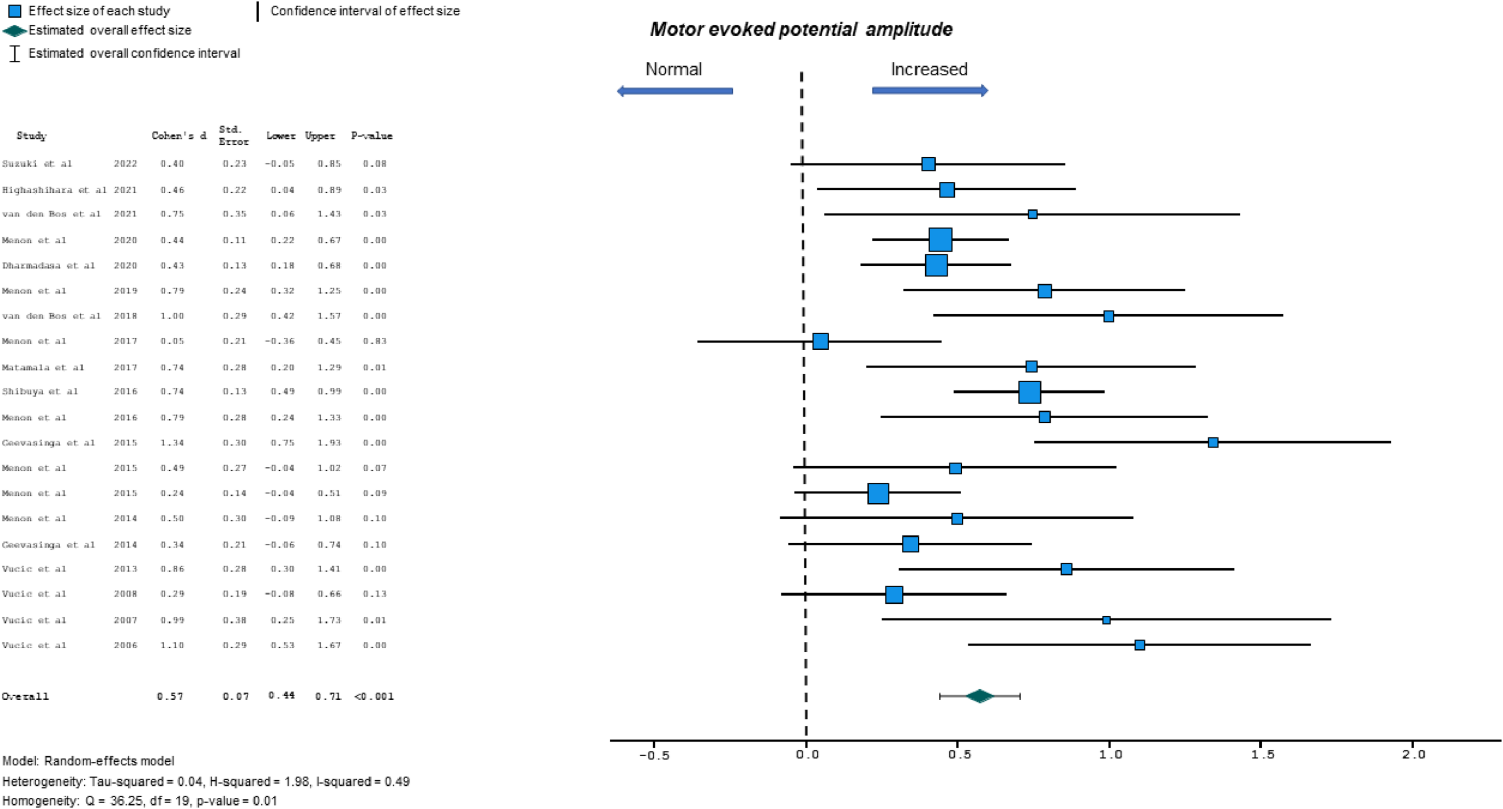
Forest plot depicting effect sizes for individual studies and an overall effect size for motor evoked potential (MEP) amplitude. The dotted line represents an absence of significant difference in MEP amplitude between amyotrophic lateral sclerosis patients and controls.

The MEP amplitude, expressed as the percentage of the compound muscle action potential response, has been increased in ALS patients signifying cortical hyperexcitability [7]. Increased MEP amplitude was significantly associated with ALS, with evidence of moderate heterogeneity (0.57, 95% CI 0.44–0.71, *p* < 0.001; *Q* = 36.25, *p* = 0.01; *I* ^2^ = 49%; Figure 5). No significant funnel plot asymmetry was evident for MEP amplitude (Egger's test slope intercept 0.09, *p* = 0.63).

Meta‐regression analysis indicated that disease duration, ALSFRS‐R, site of disease onset, UMN score or gender (proportion of males) were not significant predictors of the relationship between MEP amplitude and ALS (Table 3).

#### Resting motor threshold

**FIGURE 6:**
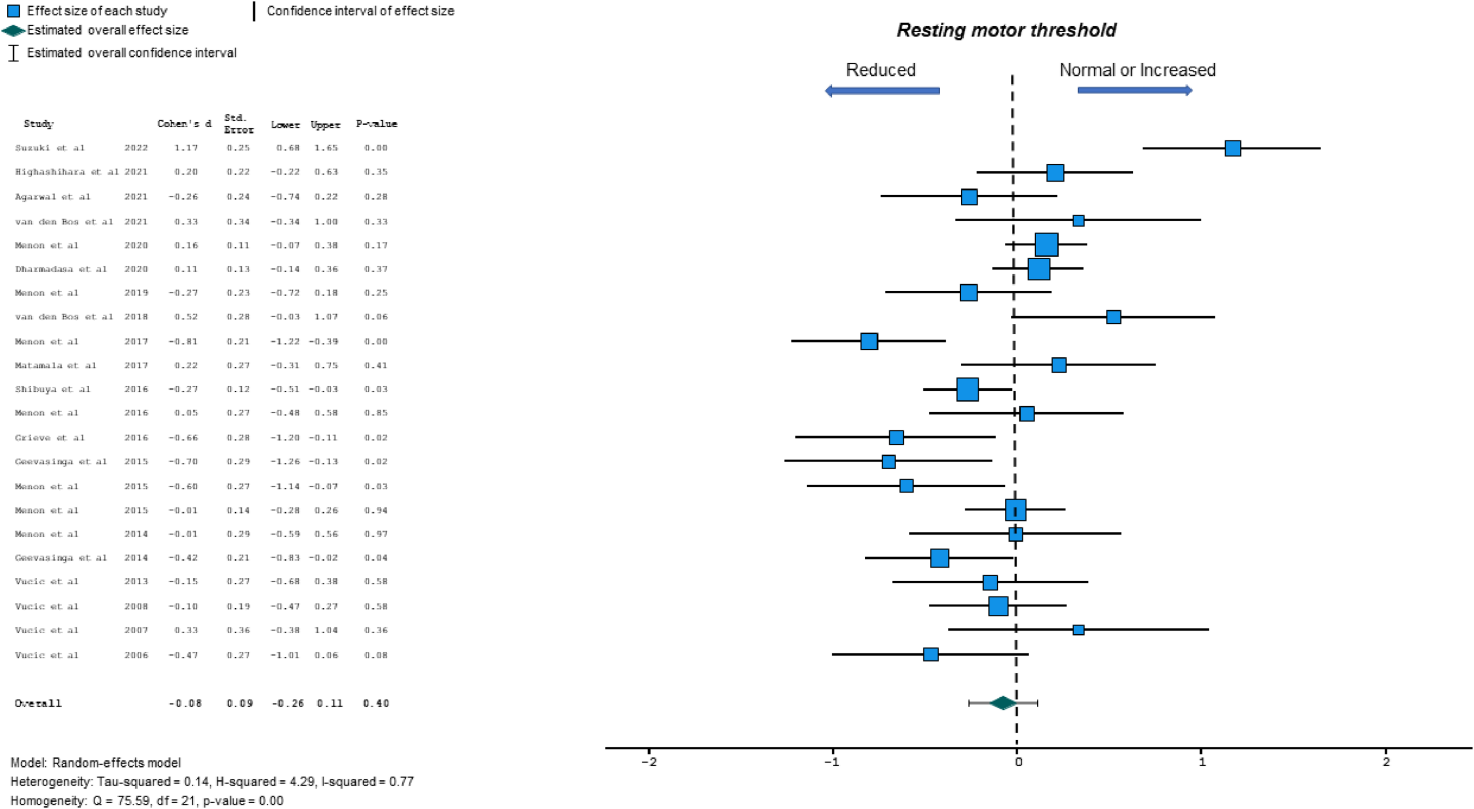
Forest plot depicting effect sizes for individual studies and an overall effect size for resting motor threshold (RMT). The dotted line represents an absence of significant difference in RMT between amyotrophic lateral sclerosis patients and controls.

Resting motor threshold (RMT) was assessed using the threshold tracking technique across all studies [14]. The SMD was −0.08 (95% CI −0.26 to 0.11, *p* = 0.40; *Q* = 75.59, *p* < 0.001; *I* ^2^ = 77%; Figure 6), suggesting that RMT poorly discriminated between ALS and controls. Significant effect size heterogeneity was evident, with effect size being markedly different in one study on Japanese ALS patients [34]. Whilst exclusion of this study increased the SMD (−0.14, 95% CI −0.28 to 0.10, *p* = 0.07; *I* ^2^ = 62%), the SMD remained non‐significant.

Meta‐regression analysis suggested that disease‐onset site was a predictor of RMT change in ALS (Table 3). Subgroup analysis disclosed that the effect size was significantly greater in studies reporting a lower (SMD_<30%_ 0.64, 95% CI 0.4–0.88) compared to those reporting a higher (SMD_≥30%_ 0.55, 95% CI −0.37 to 0.73; *I* ^2^ = 57.6%) proportion of bulbar‐onset ALS patients, suggesting that RMT was higher in limb‐onset ALS. In contrast, disease duration, ALSFRS‐R, UMN score or gender (proportion of males) were not significant predictors of the relationship between mean MEP amplitude and ALS (Table 3).

## DISCUSSION

The present meta‐analysis, incorporating over 2500 participants, established diagnostic utility of threshold tracking TMS measures of cortical hyperexcitability in differentiating ALS from controls. Reduction of mean SICI (between ISIs 1 and 7 ms) exhibited the highest SMD, suggesting that this TMS measure was the most sensitive TMS biomarker of cortical hyperexcitability in ALS, thereby exhibiting greatest diagnostic utility. The reduction of SICI was independent of disease duration, degree of functional disability, site of disease onset, gender and racial differences. Abnormalities in other TMS measures were in keeping with cortical hyperexcitability, with reduction of CSP duration and increased MEP amplitude exhibiting greater utility than ICF increase or RMT reduction. Incorporation of SICI into commercially available TMS devices could provide an important diagnostic aid, particularly in the early stages of ALS. At a pathophysiological level, the meta‐analysis indicated that motor cortical disinhibition is a more important mechanism for cortical hyperexcitability development, suggesting a proclivity for dysfunction of inhibitory interneuronal circuits and thereby focusing therapeutic strategies towards inhibitory cortical circuits.

Short interval intracortical inhibition (SICI) is a biomarker of cortical inhibitory interneuronal circuits acting via GABA_A_ receptors and exerting inhibitory effects through synaptic mechanisms, combined with axonal refractoriness and, potentially, shunting inhibition achieved by the opening of channels in proximal dendrites targeted by incoming afferents [18, 56, 57]. Cortical hyperexcitability, heralded by the reduction of SICI, has been identified as an early and intrinsic feature of ALS [2, 3, 4, 58, 59], preceding lower motor neuron dysfunction [46] and associated with an adverse prognosis [42], disease evolution [9, 11, 33] and development of clinical features including the split‐hand phenomenon [10]. Dysfunction or degeneration of the interneuronal GABAergic circuits was postulated to underlie SICI reduction in ALS [6], and thereby development of cortical hyperexcitability. Whilst SICI reduction may represent a compensatory response to lower motor neuron degeneration [2], the findings of normal cortical excitability in neuromuscular mimic disorders of ALS [4, 7, 19], as well as partial and transient normalization of SICI with institution of riluzole therapy (proposed anti‐glutaminergic agent) [43, 49], would argue against such an explanation.

In the current meta‐analysis, a significant reduction of mean SICI (between ISIs 1 and 7 ms) was evident in ALS, with a higher SMD (−0.994, 95% CI −1.12 to −0.873) compared to other TMS parameters of cortical hyperexcitability. Meta‐regression analysis suggested that disease duration, degree of functional disability, site of disease onset, gender or racial background did not significantly influence the relationship between SICI reduction and ALS. Consequently, it could be implied that SICI reduction was an early feature in ALS, consistently evident across different disease stages (as defined by disease duration and level of functional disability), as well as gender, racial background and site of disease onset. Previous studies have reported a greater reduction of SICI in ALS patients with longer disease durations (>17 months) [33, 38], suggesting a progressive dysfunction of cortical GABAergic inhibitory interneuronal circuits. A likely explanation for discordant findings may relate to shorter disease duration (median 15.3 months, range 11–17.5 months) in the current study, whilst not including patients with longer disease durations (>17 months). Underscoring this fact was the finding of a comparable SICI reduction across the King's stages of disease, in which the median disease duration was comparable in patients with disease durations of less than 17 months (stage 1, 12 months; stage 2, 10 months; and stage 3, 12 months) [38].

Reduction in CSP duration was also in keeping with the presence of cortical disinhibition in ALS and in keeping with previous studies [18]. The SMD (−0.655, 95% CI −0.808 to −0.501) was smaller compared to SICI, suggesting that CSP reduction was not as robust a biomarker of cortical hyperexcitability, a finding in keeping with previous studies [7]. The reduction of CSP duration in ALS is mediated by dysfunction of longer latency inhibitory GABA_B_ circuits as well as concomitant dysfunction spinal inhibitory circuits [60]. Alteration of CSP mediating inhibitory circuits appears to be an early feature of ALS, in keeping with a previous study, which did not evolve with disease progression or functional status [38]. The reason for significant heterogeneity of CSP duration might be multifactorial, relating to muscle fatigue during contraction and inconsistent determination of the onset of CSP. An explanation for greater utility of SICI reduction as a biomarker of cortical hyperexcitability remains to be fully elucidated, although it may reflect greater vulnerability of the shorter latency motor cortical GABAergic circuits in ALS.

An increase in cortical facilitation also contributes to the development of hyperexcitability in ALS, as evident by higher MEP amplitudes and ICF. The MEP amplitude reflects density and excitability of corticomotoneuronal projections onto spinal motor neurons, increased by adrenergic neurotransmission as well as sodium and calcium conductances [56]. ICF may appear to be mediated by distinct cortical facilitatory circuits [61] or through physiological mechanisms operating at a spinal level [62]. The increase in MEP amplitude and ICF did not change with disease duration or functional status, suggesting that these TMS changes become evident during the early stages of ALS. The SMD for MEP amplitude and ICF were smaller compared to SICI and CSP duration, suggesting that cortical inhibition was a greater contributor to development of hyperexcitability than increased facilitation, and in keeping with previous studies undertaken in smaller cohorts [7]. The discordance between measures of cortical inhibition and facilitation could be related to higher MEP amplitude variability [63] and inconsistent identification of ICF [64], suggesting an overall poorer reproducibility of these TMS measures.

Alternatively, the findings achieved through interrogation of cortical motor pathways could be related to a proclivity for dysfunction and degeneration of cortical inhibitory circuits in ALS. Specifically, dysfunction of cortical inhibitory pathways could represent a key pathophysiological mechanism that underlies neurodegeneration in ALS, heralded through the manifestation of cortical hyperexcitability and the ensuing lower motor neuron degeneration. Recent studies have identified the loss of Lamp5 (novel regulator of neuronal hyperexcitation critical for survival of distinct interneuronal populations) in Alzheimer's disease, resulting in dysfunction of cortical networks that are linked to cortical hyperexcitability and neurodegeneration [65]. Assessing for the dysfunction of Lamp5 and related modulators of cortical inhibitory interneuronal function, with a view to unlocking the initial pathogenic event in ALS, may further inform pathophysiological insights into neurological disease and lead to development of novel therapeutic strategies.

Resting motor threshold (RMT) reflects excitability of corticomotoneuronal fibres projecting onto spinal motor neurons that innervate a target muscle [18]. Interestingly, RMT was not significantly reduced in ALS patients in the current meta‐analysis, although subgroup analysis suggested a significant increase in limb‐onset patients. A possible explanation may relate to variability in RMT across different studies, reflecting clinical heterogeneity of ALS. Specifically, whilst some studies reported RMT reduction as an early feature of ALS [8, 21], others have documented normal [2, 3, 9, 21] or increased RMT [22, 23], with the latter findings reflecting UMN degeneration [21]. The effect size in one study was markedly different [34], and omission of this study from the meta‐analysis did not appreciably change the overall effect size (−0.135, 95% CI −0.284 to 0.013, *p* = 0.074; *Q* = 49.80, *p* < 0.001; *I* ^2^ = 62%). Subgroup analysis disclosed a significant effect size in limb‐onset ALS, suggesting that increased RMT is a more reliable cortical hyperexcitability biomarker in the limb‐onset group of patients. Whilst RMT may be influenced by use of neuromodulating agents, such as Na^+^ channel blockers, this did not account for the findings as none of the studies reported use of neuromodulating agents that could impact RMT values. Rather, the findings could reflect the pathophysiological heterogeneity across the ALS phenotype.

The current meta‐analysis included studies utilizing the threshold tracking TMS technique with serial ascending ordering of ISIs [14]. TMS studies utilizing the constant stimulus method were excluded due to methodological differences, rendering direct comparisons of the paired‐pulse physiological measures difficult. Additionally, the parallel threshold tracking TMS method reported highest diagnostic utility at ISIs of 2.5 ms [58, 59], contrasting with the serial ascending method where mean SICI (between ISI 1 and 7 ms) was reported to exhibit highest diagnostic utility [7]. The differences in area under the curve were subtle, at least as far as the typical ALS phenotype is concerned, and whether these were significantly different could not be determined for the parallel tracking TMS papers as the 95% CIs were not reported. Notwithstanding this, the varied findings could relate to differences in methodology, including the use of different coils (figure of eight vs. circular coil) and recording sites (first dorsal interosseous vs. abductor pollicis brevis muscle), thereby precluding direct comparisons between the parallel tracking method and studies included in the meta‐analysis. Large multicentre studies, comparing the three TMS methodologies, may be required to determine the diagnostic utility of different SICI components in ALS. It should be acknowledged that both constant stimulus [2, 66, 67, 68, 69, 70] and parallel threshold tracking [58, 59] TMS techniques have reported significant SICI reduction in ALS, in keeping with the current meta‐analysis and suggesting that similar cortical disinhibition contributed to ALS pathophysiology. Separately, it is accepted that use of aggregate data rather than individual patient data in the current meta‐analysis could have impacted the findings. Whilst equivalence of summary effects between the aggregate and individual patient data methods has been reported [71], thereby arguing against an impact of the aggregate based methodology on the findings, it should be acknowledged that the trajectory of TMS measures (SICI, ICF, MEP amplitude, CSP duration and RMT) are likely to be variable at an individual patient level, thereby impacting on diagnostic utility. An individual patient data meta‐analysis may help clarify the utility of the various TMS parameters at a patient level.

From a clinical perspective, the present study established that reduction in mean SICI (between ISIs 1 and 7 ms) exhibited the highest SMD, as assessed from a large cohort of ALS patients (>1500) and controls (>1100), implying greater clinical diagnostic utility compared to other TMS biomarkers. Integration of the paired‐pulse TMS paradigm into commercially available devices may facilitate ALS diagnosis by enabling measurements of SICI in a clinical setting, although confirmatory multicentre studies are still required. Additionally, TMS measures of cortical hyperexcitability could also serve as surrogate biomarkers of cognitive and behavioural dysfunction in ALS and ALS–frontotemporal dementia overlap syndrome [35, 36]. Combining SICI with other TMS measures, including CSP duration, MEP amplitude and ICF, could also aid in diagnostic utility, from both motor and cognitive aspects, as well as obviating confounding effects such as neuromodulating medications. If confirmed, clinical application of cortical excitability measurements may lead to earlier commencement of appropriate therapeutic agents in a multidisciplinary clinical environment, as well recruitment into clinical trials [72].

## AUTHOR CONTRIBUTIONS

**Steve Vucic:** Conceptualization; investigation; writing – original draft; visualization; validation; methodology; software; formal analysis; project administration; data curation; supervision; resources. **Mana Higashihara:** Conceptualization; investigation; writing – original draft; methodology; formal analysis; data curation. **Nathan Pavey:** Conceptualization; investigation; methodology; validation; writing – review and editing; formal analysis; software; data curation. **Parvathi Menon:** Conceptualization; writing – review and editing; resources; methodology. **Mehdi van den Bos:** Conceptualization; writing – review and editing; formal analysis; data curation. **Kazumoto Shibuya:** Conceptualization; writing – review and editing; formal analysis. **Satoshi Kuwabara:** Conceptualization; investigation; methodology; writing – review and editing. **Matthew Kiernan:** Writing – review and editing; conceptualization; supervision. **Masayoshi Koinuma:** Conceptualization; investigation; writing – review and editing; methodology; formal analysis; data curation; supervision.

## CONFLICT OF INTEREST STATEMENT

The authors declare the following funding sources that did not influence the study. PM recipient of the MND Research Australia (Betty Laidlaw Research Award) fellowship; SV recipient of the National Health and Medical Research Council ideas grant (grant no. 2010812); MCK was supported by an NHMRC Practitioner Fellowship (1156093). There are no other conflicts of interest.

## Data Availability

The data that support the findings of this study are available from the corresponding author upon reasonable request.

## References

[1] Shefner JM , Al‐Chalabi A , Baker MR , et al. A proposal for new diagnostic criteria for ALS. Clin Neurophysiol. 2020;131:1975‐1978. 32387049 10.1016/j.clinph.2020.04.005

[2] Zanette G , Tamburin S , Manganotti P , Refatti N , Forgione A , Rizzuto N . Different mechanisms contribute to motor cortex hyperexcitability in amyotrophic lateral sclerosis. Clin Neurophysiol. 2002;113:1688‐1697. 12417221 10.1016/s1388-2457(02)00288-2

[3] Vucic S , Kiernan MC . Novel threshold tracking techniques suggest that cortical hyperexcitability is an early feature of motor neuron disease. Brain. 2006;129:2436‐2446. 16835248 10.1093/brain/awl172

[4] Vucic S , Kiernan MC . Cortical excitability testing distinguishes Kennedy's disease from amyotrophic lateral sclerosis. Clin Neurophysiol. 2008;119:1088‐1096. 18313980 10.1016/j.clinph.2008.01.011

[5] Vucic S , van den Bos M , Menon P , Howells J , Dharmadasa T , Kiernan MC . Utility of threshold tracking transcranial magnetic stimulation in ALS. Clin Neurophysiol Pract. 2018;3:164‐172. 30560220 10.1016/j.cnp.2018.10.002 PMC6275211

[6] Nihei K , McKee AC , Kowall NW . Patterns of neuronal degeneration in the motor cortex of amyotrophic lateral sclerosis patients. Acta Neuropathol. 1993;86:55‐64. 8396837 10.1007/BF00454899

[7] Menon P , Geevasinga N , Yiannikas C , Howells J , Kiernan MC , Vucic S . Sensitivity and specificity of threshold tracking transcranial magnetic stimulation for diagnosis of amyotrophic lateral sclerosis: a prospective study. Lancet Neurol. 2015;14:478‐484. 25843898 10.1016/S1474-4422(15)00014-9

[8] Eisen A , Weber M . The motor cortex and amyotrophic lateral sclerosis. Muscle Nerve. 2001;24:564‐573. 11268031 10.1002/mus.1042

[9] Menon P , Geevasinga N , van den Bos M , Yiannikas C , Kiernan MC , Vucic S . Cortical hyperexcitability and disease spread in amyotrophic lateral sclerosis. Eur J Neurol. 2017;24:816‐824. 28436181 10.1111/ene.13295

[10] Menon P , Kiernan MC , Vucic S . Cortical dysfunction underlies the development of the split‐hand in amyotrophic lateral sclerosis. PLoS One. 2014;9:e87124. 24475241 10.1371/journal.pone.0087124 PMC3901749

[11] Dharmadasa T , Matamala JM , Howells J , Vucic S , Kiernan MC . Early focality and spread of cortical dysfunction in amyotrophic lateral sclerosis: a regional study across the motor cortices. Clin Neurophysiol. 2020;131:958‐966. 31959568 10.1016/j.clinph.2019.11.057

[12] Di Lazzaro V , Ranieri F , Profice P , et al. Transcranial direct current stimulation effects on the excitability of corticospinal axons of the human cerebral cortex. Brain Stimul. 2013;6:641‐643. 23085442 10.1016/j.brs.2012.09.006

[13] Kujirai T , Caramia MD , Rothwell JC , et al. Corticocortical inhibition in human motor cortex. J Physiol. 1993;471:501‐519. 8120818 10.1113/jphysiol.1993.sp019912 PMC1143973

[14] Vucic S , Howells J , Trevillion L , Kiernan MC . Assessment of cortical excitability using threshold tracking techniques. Muscle Nerve. 2006;33:477‐486. 16315324 10.1002/mus.20481

[15] Fisher RJ , Nakamura Y , Bestmann S , Rothwell JC , Bostock H . Two phases of intracortical inhibition revealed by transcranial magnetic threshold tracking. Exp Brain Res. 2002;143:240‐248. 11880900 10.1007/s00221-001-0988-2

[16] Vucic S , Kiernan MC . Abnormalities in cortical and peripheral excitability in flail arm variant amyotrophic lateral sclerosis. J Neurol Neurosurg Psychiatry. 2007;78:849‐852. 17210625 10.1136/jnnp.2006.105056 PMC2117729

[17] Geevasinga N , Menon P , Nicholson GA , et al. Cortical function in asymptomatic carriers and patients with C9orf72 amyotrophic lateral sclerosis. JAMA Neurol. 2015;72:1268‐1274. 26348842 10.1001/jamaneurol.2015.1872 PMC4707047

[18] Vucic S , Stanley Chen KH , Kiernan MC , et al. Clinical diagnostic utility of transcranial magnetic stimulation in neurological disorders. Updated report of an IFCN committee. Clin Neurophysiol. 2023;150:131‐175. 37068329 10.1016/j.clinph.2023.03.010 PMC10192339

[19] Vucic S , Nicholson GA , Kiernan MC . Cortical excitability in hereditary motor neuronopathy with pyramidal signs: comparison with ALS. J Neurol Neurosurg Psychiatry. 2010;81:97‐100. 20019225 10.1136/jnnp.2008.157537

[20] Geevasinga N , Korgaonkar MS , Menon P , et al. Brain functional connectome abnormalities in amyotrophic lateral sclerosis are associated with disability and cortical hyperexcitability. Eur J Neurol. 2017;24:1507‐1517. 28926154 10.1111/ene.13461

[21] Mills KR , Nithi KA . Corticomotor threshold is reduced in early sporadic amyotrophic lateral sclerosis. Muscle Nerve. 1997;20:1137‐1141. 9270669 10.1002/(sici)1097-4598(199709)20:9<1137::aid-mus7>3.0.co;2-9

[22] Eisen A , Shytbel W , Murphy K , Hoirch M . Cortical magnetic stimulation in amyotrophic lateral sclerosis. Muscle Nerve. 1990;13:146‐151. 2314418 10.1002/mus.880130211

[23] Urban P , Wicht S , Hopf H . Sensitivity of transcranial magnetic stimulation of cortico‐bulbar vs. cortico‐spinal tract involvement in ALS. J Neurol. 2001;248:850‐855. 11697520 10.1007/s004150170068

[24] Moher D , Liberati A , Tetzlaff J , Altman DG , PRISMA Group . Preferred reporting items for systematic reviews and meta‐analyses: the PRISMA statement. Ann Intern Med. 2009;151:264‐269. 19622511 10.7326/0003-4819-151-4-200908180-00135

[25] Tankisi H , Cengiz B , Howells J , Samusyte G , Koltzenburg M , Bostock H . Short‐interval intracortical inhibition as a function of inter‐stimulus interval: three methods compared. Brain Stimul. 2021;14:22‐32. 33166726 10.1016/j.brs.2020.11.002

[26] Shibuya K , Park SB , Geevasinga N , et al. Threshold tracking transcranial magnetic stimulation: effects of age and gender on motor cortical function. Clin Neurophysiol. 2016;127:2355‐2361. 27178853 10.1016/j.clinph.2016.03.009

[27] Shibuya K , Park SB , Howells J , et al. Laterality of motor cortical function measured by transcranial magnetic stimulation threshold tracking. Muscle Nerve. 2017;55:424‐427. 27511622 10.1002/mus.25372

[28] Vucic S , Cheah BC , Kiernan MC . Maladaptation of cortical circuits underlies fatigue and weakness in ALS. Amyotroph Lateral Scler Frontotemporal Degener. 2011;12:414‐420. 10.3109/17482968.2011.597403 21830989

[29] Vucic S , Cheah BC , Yiannikas C , Kiernan MC . Cortical excitability distinguishes ALS from mimic disorders. Clin Neurophysiol. 2011;122:1860‐1866. 21382747 10.1016/j.clinph.2010.12.062

[30] Vucic S , Cheah BC , Yiannikas C , Vincent A , Kiernan MC . Corticomotoneuronal function and hyperexcitability in acquired neuromyotonia. Brain. 2010;133:2727‐2733. 20736187 10.1093/brain/awq188 PMC2929332

[31] Vucic S , Kiernan M . Clarifying variability of corticomotoneuronal function in Kennedy's disease. Muscle Nerve. 2011;44:197‐201. 21698646 10.1002/mus.22017

[32] Vucic S , Kiernan MC . Upregulation of persistent sodium conductances in familial ALS. J Neurol Neurosurg Psychiatry. 2010;81:222‐227. 19726402 10.1136/jnnp.2009.183079

[33] Shibuya K , Simon NG , Geevasinga N , et al. The evolution of motor cortical dysfunction in amyotrophic lateral sclerosis. Clin Neurophysiol. 2017;128:1075‐1082. 28400096 10.1016/j.clinph.2017.03.004

[34] Suzuki YI , Shibuya K , Misawa S , et al. Relationship between motor cortical and peripheral axonal hyperexcitability in amyotrophic lateral sclerosis. J Neurol Neurosurg Psychiatry. 2022;93:1074‐1079. 10.1136/jnnp-2021-328550 35995552

[35] Higashihara M , Pavey N , van den Bos M , Menon P , Kiernan MC , Vucic S . Association of cortical hyperexcitability and cognitive impairment in patients with amyotrophic lateral sclerosis. Neurology. 2021;96:e2090‐e2097. 33827958 10.1212/WNL.0000000000011798

[36] Agarwal S , Highton‐Williamson E , Caga J , et al. Motor cortical excitability predicts cognitive phenotypes in amyotrophic lateral sclerosis. Sci Rep. 2021;11:2172. 33500476 10.1038/s41598-021-81612-x PMC7838179

[37] van den Bos MAJ , Higashihara M , Geevasinga N , Menon P , Kiernan MC , Vucic S . Pathophysiological associations of transcallosal dysfunction in ALS. Eur J Neurol. 2021;28:1172‐1180. 33220162 10.1111/ene.14653

[38] Menon P , Higashihara M , van den Bos M , Geevasinga N , Kiernan MC , Vucic S . Cortical hyperexcitability evolves with disease progression in ALS. Ann Clin Transl Neurol. 2020;7:733‐741. 32304186 10.1002/acn3.51039 PMC7261748

[39] Menon P , Yiannikas C , Kiernan MC , Vucic S . Regional motor cortex dysfunction in amyotrophic lateral sclerosis. Ann Clin Transl Neurol. 2019;6:1373‐1382. 31402622 10.1002/acn3.50819 PMC6689694

[40] van den Bos MAJ , Higashihara M , Geevasinga N , et al. Imbalance of cortical facilitatory and inhibitory circuits underlies hyperexcitability in ALS. Neurology. 2018;91:e1669‐e1676. 30282772 10.1212/WNL.0000000000006438

[41] Matamala JM , Geevasinga N , Huynh W , et al. Cortical function and corticomotoneuronal adaptation in monomelic amyotrophy. Clin Neurophysiol. 2017;128:1488‐1495. 28624492 10.1016/j.clinph.2017.05.005

[42] Shibuya K , Park SB , Geevasinga N , et al. Motor cortical function determines prognosis in sporadic ALS. Neurology. 2016;87:513‐520. 27402895 10.1212/WNL.0000000000002912

[43] Geevasinga N , Menon P , Ng K , et al. Riluzole exerts transient modulating effects on cortical and axonal hyperexcitability in ALS. Amyotroph Lateral Scler Frontotemporal Degener. 2016;17:580‐588. 27249331 10.1080/21678421.2016.1188961

[44] Menon P , Geevasinga N , Yiannikas C , Kiernan MC , Vucic S . Cortical contributions to the flail leg syndrome: pathophysiological insights. Amyotroph Lateral Scler Frontotemporal Degener. 2016;17:389‐396. 26888565 10.3109/21678421.2016.1145232

[45] Grieve SM , Menon P , Korgaonkar MS , et al. Potential structural and functional biomarkers of upper motor neuron dysfunction in ALS. Amyotroph Lateral Scler Frontotemporal Degener. 2015;17:1‐8. 26458122 10.3109/21678421.2015.1074707

[46] Menon P , Kiernan MC , Vucic S . Cortical hyperexcitability precedes lower motor neuron dysfunction in ALS. Clin Neurophysiol. 2015;126:803‐809. 25227219 10.1016/j.clinph.2014.04.023

[47] Geevasinga N , Menon P , Yiannikas C , Kiernan MC , Vucic S . Diagnostic utility of cortical excitability studies in amyotrophic lateral sclerosis. Eur J Neurol. 2014;21:1451‐1457. 24698287 10.1111/ene.12422

[48] Bae JS , Menon P , Mioshi E , Kiernan MC , Vucic S . Cortical hyperexcitability and the split‐hand plus phenomenon: pathophysiological insights in ALS. Amyotroph Lateral Scler Frontotemporal Degener. 2014;15:250‐256. 24555863 10.3109/21678421.2013.872150

[49] Vucic S , Lin CS‐Y , Cheah BC , et al. Riluzole exerts central and peripheral modulating effects in amyotrophic lateral sclerosis. Brain. 2013;136:1361‐1370. 23616585 10.1093/brain/awt085

[50] Cedarbaum JM , Stambler N , Malta E , et al. The ALSFRS‐R: a revised ALS functional rating scale that incorporates assessments of respiratory function. BDNF ALS Study Group (Phase III). J Neurol Sci. 1999;169:13‐21. 10540002 10.1016/s0022-510x(99)00210-5

[51] Turner M , Cagnin A , Turkheimer F , et al. Evidence of widespread cerebral microglial activation in amyotrophic lateral sclerosis: an [11C](R)‐PK11195 positron emission tomography study. Neurobiol Dis. 2004;15:601‐609. 15056468 10.1016/j.nbd.2003.12.012

[52] Egger M , Davey Smith G , Schneider M , Minder C . Bias in meta‐analysis detected by a simple, graphical test. BMJ. 1997;315:629‐634. 9310563 10.1136/bmj.315.7109.629 PMC2127453

[53] Duval S , Tweedie R . Trim and fill: a simple funnel‐plot‐based method of testing and adjusting for publication bias in meta‐analysis. Biometrics. 2000;56:455‐463. 10877304 10.1111/j.0006-341x.2000.00455.x

[54] Afolabi MO , Ale BM , Dabira ED , et al. Malaria and helminth co‐infections in children living in endemic countries: a systematic review with meta‐analysis. PLoS Negl Trop Dis. 2021;15:e0009138. 33600494 10.1371/journal.pntd.0009138 PMC7924789

[55] Yu DD , You LZ , Huang WQ , et al. Effects of traditional Chinese exercises on blood glucose and hemoglobin A1c levels in patients with prediabetes: a systematic review and meta‐analysis. J Integr Med. 2020;18:292‐302. 32534937 10.1016/j.joim.2020.04.003

[56] Ziemann U , Reis J , Schwenkreis P , et al. TMS and drugs revisited 2014. Clin Neurophysiol. 2015;126:1847‐1868. 25534482 10.1016/j.clinph.2014.08.028

[57] Suzuki YI , Ma Y , Shibuya K , et al. Effect of racial background on motor cortical function as measured by threshold tracking transcranial magnetic stimulation. J Neurophysiol. 2021;126:840‐844. 34406906 10.1152/jn.00083.2021

[58] Tankisi H , Nielsen CS , Howells J , et al. Early diagnosis of amyotrophic lateral sclerosis by threshold tracking and conventional transcranial magnetic stimulation. Eur J Neurol. 2021;28:3030‐3039. 34233060 10.1111/ene.15010 PMC9291110

[59] Tankisi H , Pia H , Strunge K , et al. Three different short‐interval intracortical inhibition methods in early diagnosis of amyotrophic lateral sclerosis. Amyotroph Lateral Scler Frontotemporal Degener. 2023;24:139‐147. 35899374 10.1080/21678421.2022.2101926

[60] Rossini PM , Burke D , Chen R , et al. Non‐invasive electrical and magnetic stimulation of the brain, spinal cord, roots and peripheral nerves: basic principles and procedures for routine clinical and research application. An updated report from an I.F.C.N. Committee. Clin Neurophysiol. 2015;126:1071‐1107. 25797650 10.1016/j.clinph.2015.02.001 PMC6350257

[61] Di Lazzaro V , Pilato F , Dileone M , et al. GABA_A_ receptor subtype specific enhancement of inhibition in human motor cortex. J Physiol. 2006;575:721‐726. 16809358 10.1113/jphysiol.2006.114694 PMC1995685

[62] Di Lazzaro V , Rothwell JC . Corticospinal activity evoked and modulated by non‐invasive stimulation of the intact human motor cortex. J Physiol. 2014;592:4115‐4128. 25172954 10.1113/jphysiol.2014.274316 PMC4215763

[63] Kiers L , Cros D , Chiappa KH , Fang J . Variability of motor potentials evoked by transcranial magnetic stimulation. Electroencephalogr Clin Neurophysiol. 1993;89:415‐423. 7507428 10.1016/0168-5597(93)90115-6

[64] van den Bos MAJ , Menon P , Howells J , et al. Physiological processes underlying short interval intracortical facilitation in the human motor cortex. Front Neurosci. 2018;12:240. 29695952 10.3389/fnins.2018.00240 PMC5904283

[65] Deng Y , Bi M , Delerue F , et al. Loss of LAMP5 interneurons drives neuronal network dysfunction in Alzheimer's disease. Acta Neuropathol. 2022;144:637‐650. 35780436 10.1007/s00401-022-02457-w PMC9467963

[66] Hanajima R , Ugawa Y , Terao Y , Ogata K , Kanazawa I . Ipsilateral cortico‐cortical inhibition of the motor cortex in various neurological disorders. J Neurol Sci. 1996;140:109‐116. 8866435 10.1016/0022-510x(96)00100-1

[67] Yokota T , Yoshino A , Inaba A , Saito Y . Double cortical stimulation in amyotrophic lateral sclerosis. J Neurol Neurosurg Psychiatry. 1996;61:596‐600. 8971106 10.1136/jnnp.61.6.596 PMC486653

[68] Ziemann U , Winter M , Reimers CD , Reimers K , Tergau F , Paulus W . Impaired motor cortex inhibition in patients with amyotrophic lateral sclerosis. Evidence from paired transcranial magnetic stimulation. Neurology. 1997;49:1292‐1298. 9371911 10.1212/wnl.49.5.1292

[69] Sommer M , Tergau F , Wischer S , Reimers CD , Beuche W , Paulus W . Riluzole does not have an acute effect on motor thresholds and the intracortical excitability in amyotrophic lateral sclerosis. J Neurol. 1999;246 Suppl 3:III22‐III26. 10631657 10.1007/BF03161086

[70] Stefan K , Kunesch E , Benecke R , Classen J . Effects of riluzole on cortical excitability in patients with amyotrophic lateral sclerosis. Ann Neurol. 2001;49:536‐539. 11310635

[71] Lyman GH , Kuderer NM . The strengths and limitations of meta‐analyses based on aggregate data. BMC Med Res Methodol. 2005;5:14. 15850485 10.1186/1471-2288-5-14 PMC1097735

[72] Kiernan MC , Vucic S , Talbot K , et al. Improving clinical trial outcomes in amyotrophic lateral sclerosis. Nat Rev Neurol. 2020;17:1‐15. 10.1038/s41582-020-00434-z PMC7747476 33340024

